#  Testes-specific hemoglobins in *Drosophila *evolved by a combination of sub- and neofunctionalization after gene duplication

**DOI:** 10.1186/1471-2148-12-34

**Published:** 2012-03-19

**Authors:** Eva Gleixner, Holger Herlyn, Stefan Zimmerling, Thorsten Burmester, Thomas Hankeln

**Affiliations:** 1 Institute of Molecular Genetics, University of Mainz, 55099 Mainz, Germany; 2 Center for Systems Biology, University of Freiburg, 79104 Freiburg, Germany; 3 Institute of Anthropology, University of Mainz, 55099 Mainz, Germany; 4 Biocenter Grindel and Zoological Museum, University of Hamburg, 20146 Hamburg, Germany

## Abstract

**Background:**

For a long time the presence of respiratory proteins in most insects has been considered unnecessary. However, in recent years it has become evident that globins belong to the standard repertoire of the insect genome. Like most other insect globins, the *glob1 *gene of *Drosophila melanogaster *displays a conserved expression pattern in the tracheae, the fat body and the Malpighian tubules.

**Results:**

Here we show that the recently discovered *D. melanogaster *globin genes *glob2 *and *glob3 *both display an unusual male-specific expression in the reproductive tract during spermatogenesis. Both paralogs are transcribed at equivalent mRNA levels and largely overlap in their cellular expression patterns during spermatogenesis. Phylogenetic analyses showed that *glob2 *and *glob3 *reflect a gene duplication event that occurred in the ancestor of the *Sophophora *subgenus at least 40 million years ago. Therefore, flies of the *Drosophila *subgenus harbor only one *glob2/3*-like gene.

**Conclusions:**

Phylogenetic and sequence analyses indicate an evolution of the *glob2 *and *glob3 *duplicates by a combination of sub- and neofunctionalization. Considering their restricted, testes-specific expression, an involvement of both globins in alleviating oxidative stress during spermatogenesis is conceivable.

## Background

O_2 _supply in insects is mainly accomplished by the highly specialized and effective tracheal system. Respiratory proteins like hemoglobins (Hbs) or hemocyanins had long been considered dispensable in insects [1,2]. Only a few taxa that are specifically adapted to a hypoxic environment were considered as exceptions [3]. Among these, the larvae of the horse botfly *Gasterophilus intestinalis *and backswimmers of the genus *Anisops *possess intracellular Hbs, which probably carry out myoglobin-like O_2_-storage functions [3-6]. Extracellular Hbs for oxygen transport and storage are present in the hemolymph of the hypoxia-tolerant aquatic larvae of chironomid midges [7,8].

In recent years, genomic sequence data have provided evidence that *Hb *genes are indeed a standard component of the insect genome [9]. Recent discoveries of Hbs in insects include the honeybee *Apis mellifera *[10], the mosquitoes *Anopheles gambiae *and *Aedes aegypti *[11], and other dipteran, lepidopteran, coleopteran and hymenopteran species [9,12]. The insect model organism *Drosophila melanogaster *was initially shown to possess an *Hb *gene named *glob1 *(CG9734) [13]. *Glob1 *is predominantly expressed at substantial amounts in the fat body and the tracheal system of *Drosophila *embryos, larvae and adults [14]. These expression sites, which appear to be conserved features of Hbs in other insect species as well, suggest that glob1 function is associated with oxidative metabolism [9,14]. The intracellular glob1 protein binds O_2 _at high affinities (P_*50*_(O_2_) = 0.12-0.15 Torr) and forms a typical globin fold, in which the heme iron atom is hexacoordinated [14,15]. The *glob1 *gene is downregulated upon experimental hypoxia *in vitro *and *in vivo *[16,17], while hyperoxia and intermittent hypoxic regimes trigger a slight transcriptional upregulation. These data indirectly suggest that glob1 might also be instrumental in binding excess O_2 _or noxious reactive oxygen species (ROS) in the tracheal system [17].

More recently, two additional globin genes referred to as *glob2 *(CG15180) and *glob3 *(CG14675) were identified in the *Drosophila *genome [18]. These two genes represent related paralogous copies, which in *D. melanogaster *both reside on chromosome 3R about 800 kb apart in head-to-tail orientation. Phylogenetic analyses showed that *glob2 *and *glob3 *are only distantly related to *glob1 *and most other insect Hb genes [18]. The basal position of *glob2 *and *3 *in the insect Hb phylogenetic tree and the monophyly of the two duplicates were further substantiated by their exon-intron pattern [18].

Conceptual translations of *D. melanogaster glob2 *and g*lob3 *result in proteins of 222 and 195 amino acids, respectively, thus exceeding the typical globin length of about 140-150 amino acids due to N- and C-terminal extensions. In the globin fold, however, amino acid residues functionally important for heme and ligand binding (e.g. the PheCD1 and the proximal and distal histidines E7 and F8) are well conserved in the proteins. In our initial analysis [18], *glob2 *appeared to be expressed at a much lower level than *glob1 *as evidenced by only a few corresponding expression sequence tag (EST) entries in databases, while *glob3 *lacked EST transcriptional evidence. To study the functional roles of glob2 and 3 we now conducted a more detailed expression analysis of both genes across *Drosophila *developmental stages, gender and tissues. In addition, the availability of completely sequenced *Drosophila *genomes representing nine species of the *Sophophora *subgenus and three species of the *Drosophila *subgenus [19] allowed us to gain novel insight into the molecular evolution and phylogeny of the *glob2 *and *glob3 *paralogs, which is relevant for functional interpretations.

## Results

### Genomic organization of *glob2 *and *glob3 *paralogs in *Drosophila*

**Figure 1:**
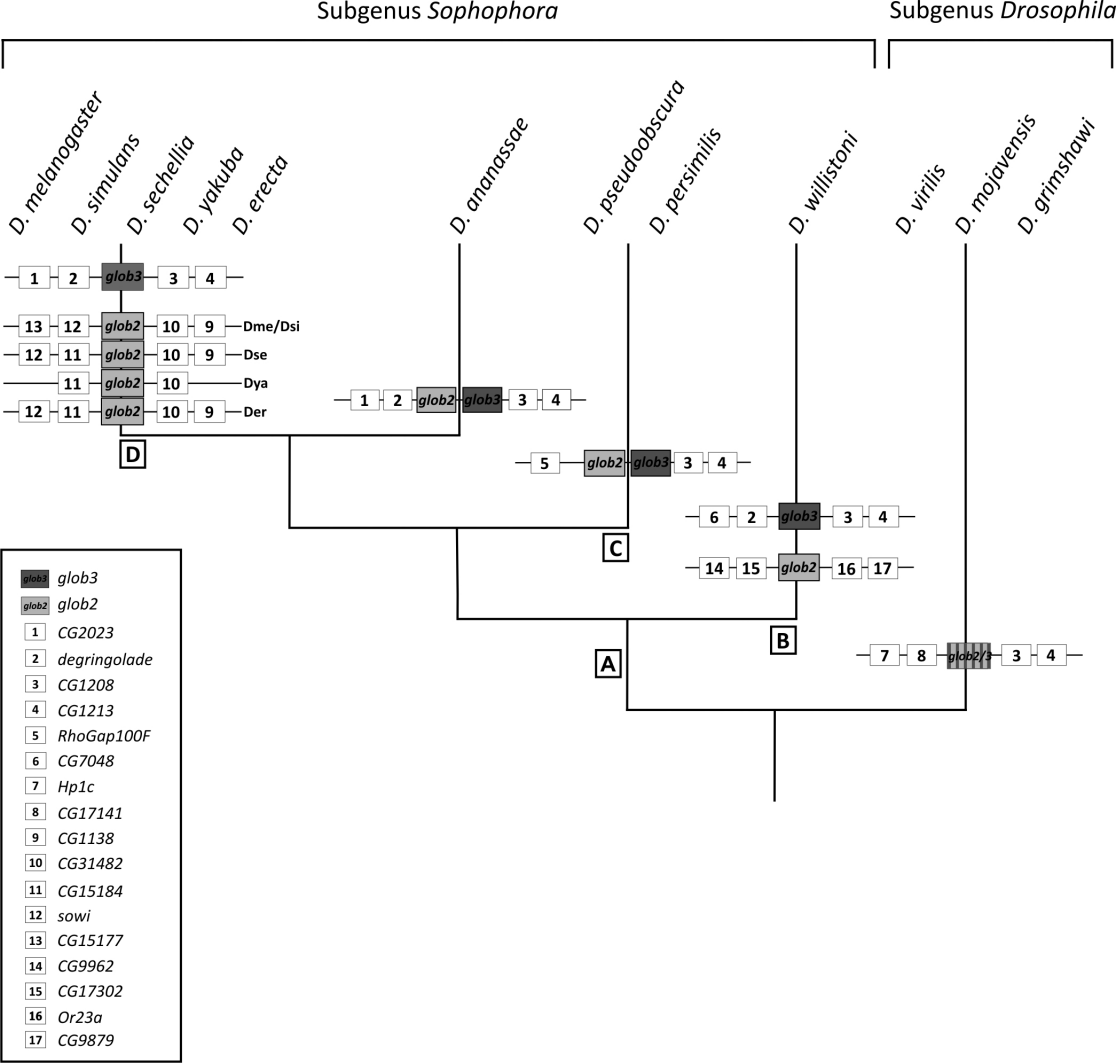
**Hypothetical scenario of evolutionary events leading to *glob2 *and *glob3 *genes in drosophilids**. The chromosomal localization and organisation of *glob2, glob3 *and *glob2/3 *genes and flanking regions in different subgroups of *Drosophila *phylogeny are displayed. [A] tandem duplication of *glob2/3 *gene; [B] separation of *glob2 *and *glob 3 *by transposition of *glob 2 *to Chr 2R (= *Dme *Chr 2L); [C] tandem *glob2 *and *glob3*, but chromosome breakage distal to the *glob2-glob3 *tandem; [D] transposition of *glob2 *to 3R 83 F4, 800 kb apart from *glob*3.

While the *glob2 *and *glob3 *gene pair is present in the nine *Drosophila *species belonging to the *Sophophora *subgenus, the three species belonging to the *Drosophila *subgenus harbour only one co-ortholog, which we name *glob2/3 *to reflect the uncertain orthology relationship (Figure 1; for gene designations, see Additional File 1). The genes located 5' of *glob3 *and *glob2/3 *vary across the investigated *Drosophila *species. In contrast, the 3' genomic environment of *glob3 *and *glob2/3 *is conserved throughout the taxon sample. In all twelve fly genomes studied *glob3 *and *glob2/3 *are located on the Muller element E, which is equivalent to chromosome 3R in *D. melanogaster*. Chromosomal location and synteny relationships are more complex for *glob2*: *D. ananassae *and *D. pseudoobscura/persimilis *harbor *glob2 *and *glob3 *as tandem duplicates in head-to-tail orientation with about 500 bp space between the gene copies. This *glob2-glob3 *tandem resides on a chromosome arm, which is equivalent to arm 3R in *D. melanogaster *(Figure 1). In *D. willistoni*, which branches off even closer to the base of the *Sophophora *subgenus, the *glob2 *copy is located on arm 2R corresponding to 2 L in *D. melanogaster *(Figure 1). In the representatives of the *D. melanogaster *subgroup, *glob2 *and *glob3 *are linked on arm 3R, but separated by about 800 kb.

### Reconstruction of a globin phylogenetic tree

Pairwise sequence comparisons of nucleotide sequences using Geneconv did not detect segments of high similarity in the complete dataset comprising 35 globin sequences. Thus subsequent analyses should not be impaired by concerted sequence evolution of paralogs within species. ML based tree reconstruction revealed a monophyletic origin of *glob2 *and *glob3 *sequences of the genus *Drosophila *(LR-ELW 99) under exclusion of *glob1 *sequences (Additional File 2A). Within this clade, *Sophophora glob2 *and *glob3 *each constitute a monophylum with moderate support (LR-ELW 83) under exclusion of *glob2/3 *from the *Drosophila *subgenus. Among the representatives of the *Sophophora *subgenus, the observed phylogeny largely corresponds to the accepted species phylogeny introduced by Clark et al. [19]. Bayesian inference essentially confirmed the ML based phylogeny, although the support for a monophyletic *Sophophora glob2-glob3 *clade was low (Additional File 2B).

Branch lengths estimated by ML and Bayesian tree inference consistently illustrate that *glob1 *orthologs diverged with lower nucleotide substitution rates than the *glob2 *and *glob 3 *orthologs. This conclusion is supported by amino acid substitution rates inferred separately for the glob1, glob2 and glob3 clades from pairwise within-group sequence comparisons using a PAM substitution matrix and assuming the *Drosophila *divergence times suggested by Tamura et al. [20]. In doing so, we calculated an average of 0.95*10^-9 ^replacements per site per year for the glob1 clade and elevated rates of 2.8*10^-9 ^and 3.5*10^-9 ^replacements per site per year for the glob2 clade and glob3 clade, respectively. Within the glob2 and 3 clades, branch length estimates and results from molecular evolutionary sequence analysis (see below, LRTII) further suggest that *Sophophora *glob 2 is more derived than *Sophophora *glob 3, compared to the hypothetical ancestor of both gene lineages.

Parametric bootstrap testing as implemented in Treefinder identified a '*glob2/3 *duplication-in-*Sophophora *subgenus' topology as the significantly better fit (p = 0.96) of the data, compared to an alternative tree topology reflecting a '*glob2 *or *3 *deletion-in-*Drosophila *subgenus' scenario.

### Molecular evolution

Due to consistent results from unconstraint tree reconstruction and hypotheses testing, we took the topology reflecting the '*glob2/3 *duplication-in-*Sophophora *subgenus' scenario as template for subsequent analyses of globin sequence evolution. We thereby focused on the evolutionary fate of *Sophophora glob2 *and *Sophophora glob3 *after the presumed duplication of an ancestral *glob2/3 *gene in the *Sophophora *stem lineage. In order to minimize any potential confounding effect of mutational saturation on the results, we addressed this issue based on a restricted sample comprising three *Drosophila glob2/3*, nine *Sophophora glob2*, and nine *Sophophora glob3 *sequences (altogether 21 sequences).

**Figure 2:**
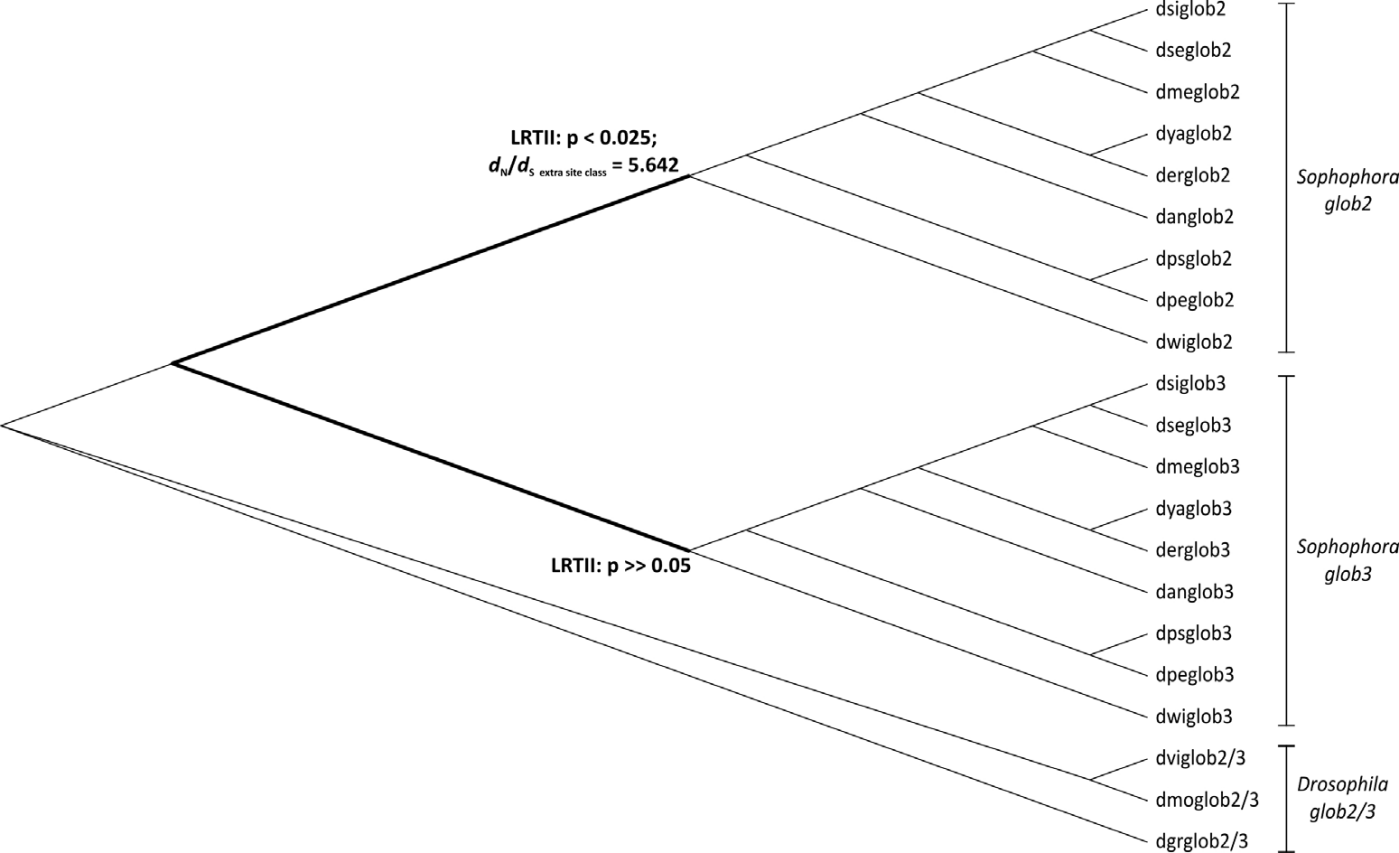
**Detection of branch-specific positive selection**. Maximum likelihood approach applying CODEML for the detection of signs of positive selection along lineages, assuming the '*glob2/3 *duplication-in-*Sophophora *subgenus' topology. An elevated omega ratio (*d*_N_/*d*_S_) was found along the branch leading to *Sophophora *glob2.

The fit of alternative beta model M8 on the sequence dataset was identical, irrespective of the initial *d*_N_/*d*_S _value chosen (*l *= - 4,398.493). As the fit of the beta null model M7 was in the same range (*l *= - 4,398.492), LRTI did not support the presence of positively selected sites across the sequence dataset (2 Δ*l *= 0.002; df = 2; cv_5% _= 5.992; p > > 0.05). The prevalence of negative purifying selection was reflected by an M7 estimate for *d*_N_/*d*_S _of 0.141. Likewise, the alternative version of modified model A (*l *= - 4,451.778) and the null implementation of modified model A (*l *= - 4,451.808) explained the evolution of the dataset equally well, when specifying the *Sophophora glob3 *stem lineage as foreground. Consequently, LRTII did not support the presence of a positively selected extra site class across the *Sophophora glob3 *stem lineage (2 Δ*l *= 0.060; df = 1; cv_5% _= 2.71; p > > 0.05). However, the alternative version of modified model A was the significantly better fit (*l *= - 4,444.314), compared to the null version (*l *= - 4,446.723), when specifying the *Sophophora glob2 *stem lineage as foreground (LRTII: 2 Δ*l *= 4.818; df = 1; cv_2.5% _= 3.84; p < 0.025). This result thus even holds when lowering the 5% level of significance to 2.5% in order to correct for twofold testing. According to the alternative version of modified model A, a remarkably high proportion of ca. 42% of codon sites underwent positive selection (*d*_N_/*d*_S _= 5.642) along the *Sophophora glob2 *stem lineage, but experienced negative selection or evolved neutrally across the background represented by all other branches of the phylogeny shown in Figure 2.

### Quantification of *glob2, glob3 *and *glob2/3 *mRNA expression

In our earlier study, six EST entries in the database of *D. melanogaster *revealed that *glob2 *is actually expressed, albeit at a rather low level [18]. Seven additional *D. melanogaster *ESTs can now be reported (UniGene:EC252960, UniGene:EL878213, UniGene:EL878330, UniGene:EL878331, UniGene:EL878455, UniGene:EC067562, UniGene:EC061683), most of which are derived from adult flies. ESTs corresponding to *glob2 *are also present for *D. simulans, D. sechellia *and *D. yakuba *adults, and eight *D. simulans *ESTs derive from 3^rd ^instar larvae.

Because of the absence of *glob3 *EST entries for *D. melanogaster *and due to our initial failure to recover cDNA from larval or adult RNA by RT-PCR we (erroneously) assumed that *glob3 *is not expressed [18]. Since 2007, however, there are three *glob3 *ESTs reported for adult *D. melanogaster *(UniGene:EL876979, UniGene:EL877168, UniGene:EL877169). In addition, we could identify three ESTs from *D. simulans *(two from an adult, one from 3^rd ^instar larvae), three ESTs from adult *D. sechellia *and one EST from adult *D. yakuba*. Based on these findings, we systematically re-analyzed *glob2, glob3 *and *glob2/3 *mRNA expression by quantitative real-time RT-PCR in embryos, male and female larvae, pupae and adult males/females of *D. melanogaster *and the distantly related *D. virilis*.

**Figure 3:**
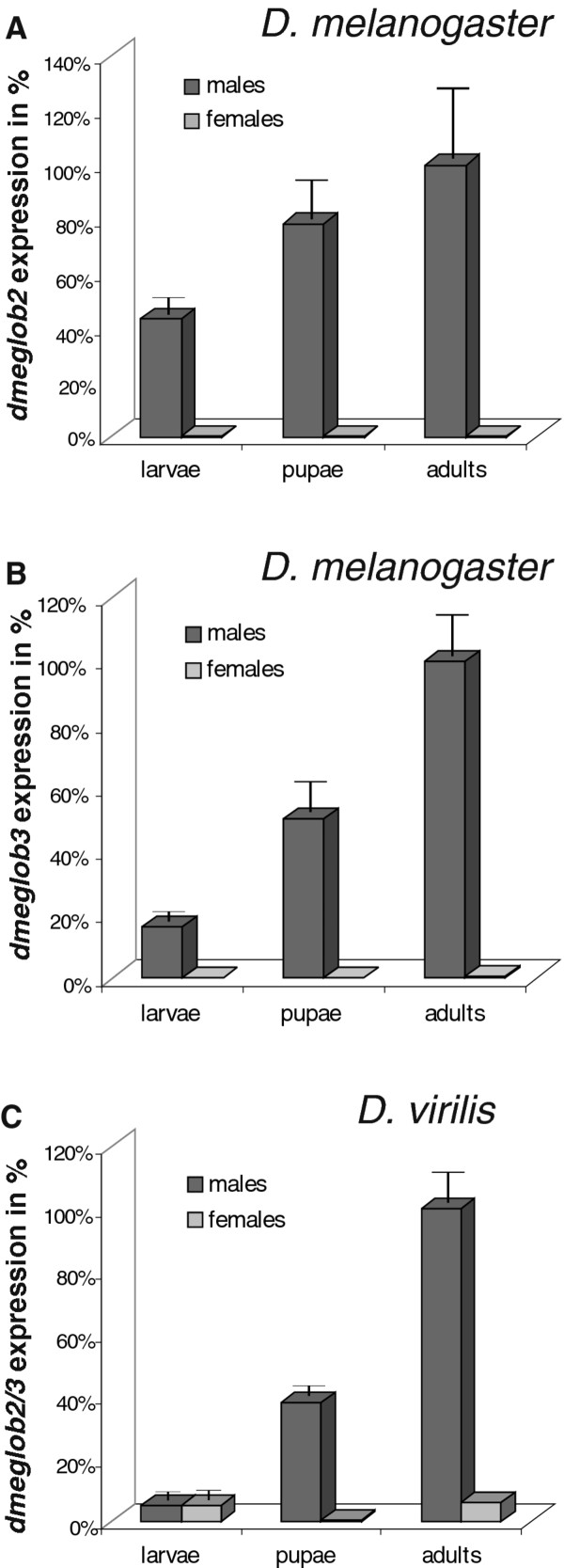
***Dmeglob2, dmeglob3 *and *dviglob2/3 *mRNA expression pattern**. Quantification of mRNA expression of *glob2 *and *glob3 *in *D. melanogaster *and *glob2/3 *in *D. virilis *in sexed 3^rd ^instar larvae, pupae and adults. mRNA levels (bars) are shown relative to *glob*-expression in adult males. (A) *glob2 *expression in *D. melanogaster*. (B) *glob3 *expression in *D. melanogaster*. (C) *glob2/3 *expression in *D. virilis*.

In embryos, the mRNA expression of *D. melanogaster glob2 *was minimal (not shown). *Dmeglob2 *turned out to be maximally expressed in male adult flies (Figure 3A). Expression levels in male larvae and pupae were at 43% and 78% relative to male adults. In female larvae, pupae and adults *dmeglob2 *expression was always beyond the detection limit. The developmental expression pattern of *dmeglob3 *showed a high similarity to *dmeglob2. Dmeglob3 *mRNA expression in embryos (not shown) as well as in female larvae, pupae and adult flies was not detectable. The amounts of *dmeglob3 *mRNA in male larvae and pupae were about 16% and 50% of the maximal mRNA expression level obtained in male adult flies (Figure 3B). Thus, for both, *dmeglob2 *and *glob3*, we could experimentally confirm the expression pattern derived from the modENCODE project implemented in FlyBase http://flybase.org/reports/FBgn0037385.html; http://flybase.org/reports/FBgn0250846.html. The developmental expression pattern of the single-copy *glob2/3 *gene in *D. virilis *was generally matching those of the *D. melanogaster *paralogs. In embryos, no expression of *dviglob2/3 *expression could be measured (not shown), while adult males showed maximum expression (Figure 3C). Male and female larvae expressed 5%, male pupae 38%, and female pupae and adults 2% and 6% of the male adult level (Figure 3C).

We also compared absolute mRNA copy numbers and observed that *D. melanogaster glob2 *and *glob3 *expression in male adults was about the same level, with 144784 copies and 150026 copies of mRNA (per 50 ng total RNA), respectively. The amount of *D. virilis glob2/3 *mRNA (311673 mRNA copies per 50 ng total RNA) in male adult flies was approximately equivalent to the sum of the *D. melanogaster glob2 *and *glob3 *mRNA copy numbers (Additional File 3).

With the analyses of the genomic region of *D. melanogaster glob3*, we identified a non-LTR-retrotransposable element (TE) of the Jockey family 234 bp upstream of the coding sequence of *dmeglob3*. The TE has a length of about 3.1 kb and is truncated at the 5'end. It is located on the minus-strand, its 3'end exhibits an A-rich stretch and it is specific for *D. melanogaster*. By applying genomic PCR on different wild type strains of *D. melanogaster *(Oregon R, Canton S, Berlin and Acharron), we confirmed the fixation of the transposon in these wild-type strains. By comparison with genomic data from *D. sechellia*, we found that the retrotransposon ends 142 bp upstream of the 5'UTR of *dmeglob3 *(Additional File 4). To find out if the retrotransposon interferes with the promoter of *dmeglob3*, we predicted possible transcription start sites applying the hidden Markov model-based program McPromoter [21,22] and the time-delayed neuron network based program NNPP [23,24]. Only the latter identified a candidate Pol II promoter for *D. simulans glob3 *and *D. sechellia glob3 *(but not for *D. melanogaster *g*lob2 *and *glob3)*, which is located 216 bp upstream of the translation start-site and 124 bp upstream of the 5'UTR (of *dmeglob3*) spanning the corresponding integration site of the TE in *D. melanogaster *(Additional File 4).

### Tissue expression patterns of *glob2, glob3 *and *glob2/3*

**Figure 4:**
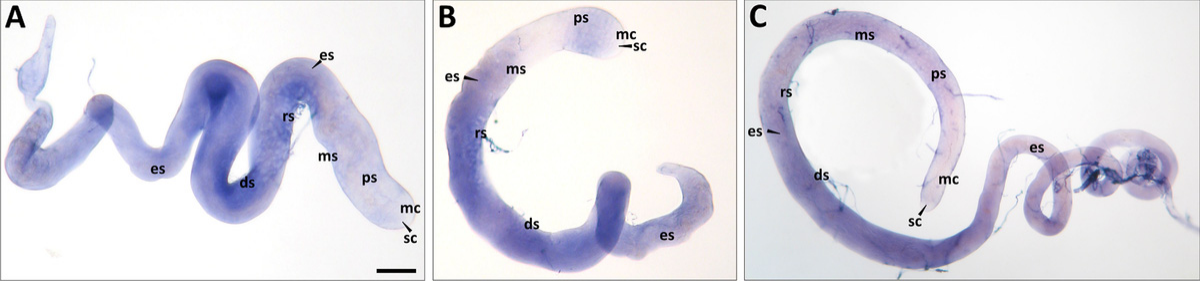
**Localization of *dmeglob2, dmeglob3 *and *dviglob2/3 *mRNA**. mRNA *in situ *hybridization of *Drosophila *adult testes with *glob2, glob3 *and *glob2/3 *antisense RNA-probes. Arrows indicate stained regions. (A) *glob2 *expression in *D. melanogaster*. (B) *glob3 *expression in *D. melanogaster*. (C) *glob2/3 *expression in *D. virilis*. All three globins analyzed show expression in primary spermatocytes, meiotic spermatocytes, round spermatids and early differentiating spermatids. Abbreviations: (sc) stem cells; (mc) mitotic cells; (ps) primary spermatocytes; (ms) meiotic spermatocytes; (rs) round spermatids; (ds) early differentiating spermatids; (es) late elongating spermatids.

The expression patterns of *glob2 *and *glob3 *were examined in head, thorax and abdomen of *D. melanogaster *adult flies by mRNA *in situ *hybridization. In head and thorax, no staining could be observed for both globin genes (data not shown). In the abdomen, the only tissues stained were the testes from male flies, as already expected from the male expression levels in qPCR. The hybridization experiments were repeated on dissected testes of *D. melanogaster *and *D. virilis*. Staining signals indicated a *glob2 *expression in various regions of the *D. melanogaster *adult testes, corresponding to several stages of spermatogenesis. Hybridization signals were observed for primary spermatocytes, meiotic spermatocytes, round spermatids and early differentiating spermatids (Figure 4A). No staining was obtained in stem cells, mitotic cells, late elongating spermatids and mature sperm. *Glob3 *in *D. melanogaster *exhibited a very similar expression pattern, although with a slightly weaker staining intensity (Figure 4B). In *D. virilis, in situ *hybridization with a *glob2/3 *antisense probe also resulted in very similar signals in primary spermatocytes, meiotic spermatocytes, round spermatids and early differentiating spermatids (Figure 4C). Sense probes, used as a negative control, did not show staining signal (Additional File 5).

### Regulation of *glob2 *and *glob3 *expression under hypoxia and hyperoxia

*D. melanogaster glob2 *and *glob3 *mRNA levels were measured by qPCR in normoxic (21% O_2_) and experimentally hypoxic as well as hyperoxic male adult *D. melanogaster. LDH*, analyzed as a positive control for hypoxia up-regulation via the HIF-1 pathway [25], showed the expected increase in mRNA expression (Additional File 6A+B).

**Figure 5:**
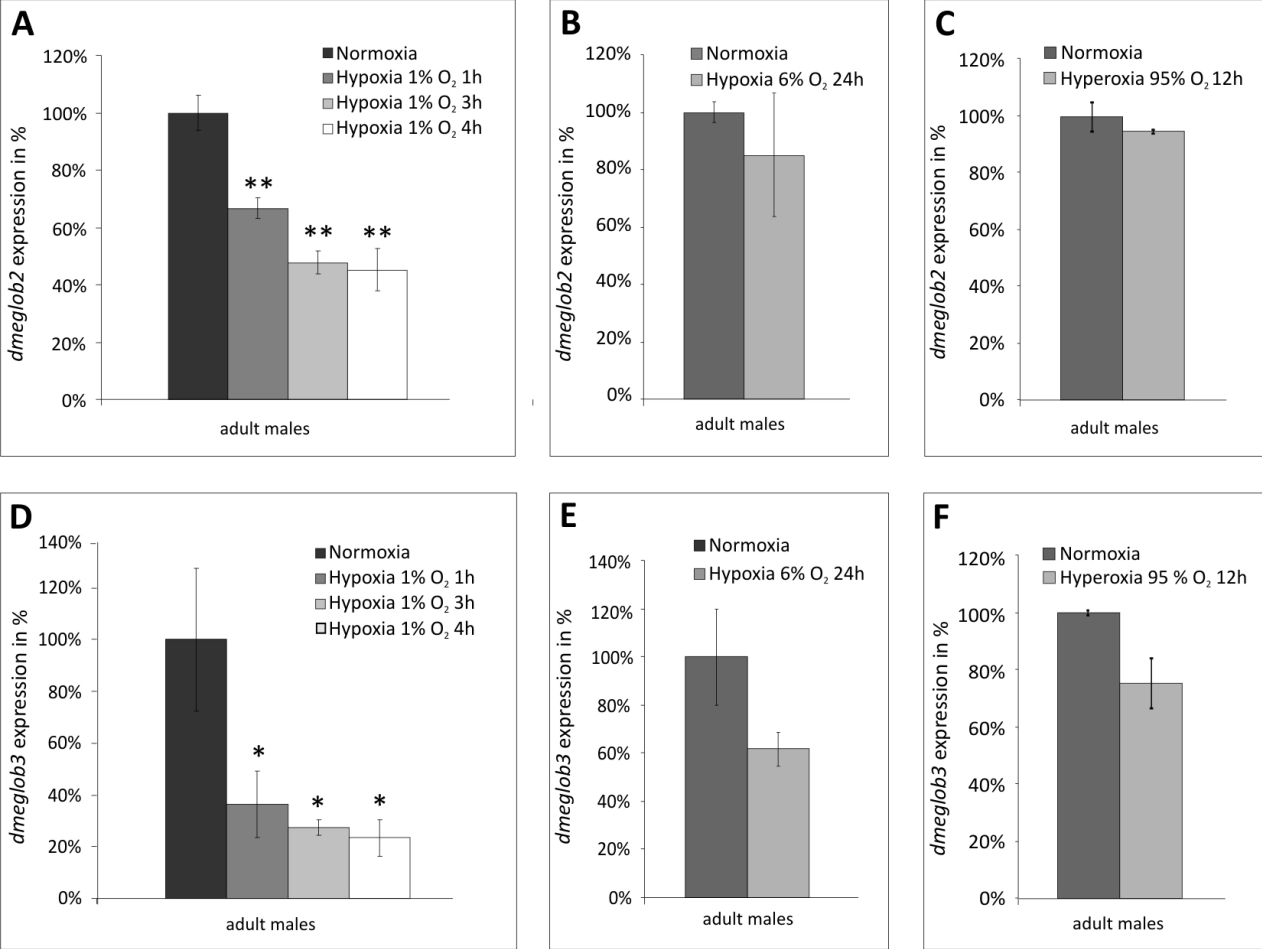
**Regulation of *dmeglob2 *and *dmeglob3 *mRNA male adults after hypoxic and hyperoxic stress**. mRNA levels (bars) are shown relative to the gene expression at normoxia (21%). (A) *Dmeglob2 *expression after 1% O_2 _for 1 h, 3 h and 4 h. (B) *Dmeglob2 *expression after 6% O_2 _for 24 h. (C) *Dmeglob3 *expression after 95% O_2 _for 12 h. (D) *Dmeglob3 *expression after 1% O_2 _for 1 h, 3 h and 4 h. (E) *Dmeglob3 *expression after 6% O_2 _for 24 h. (F) *Dmeglob3 *expression after 95% O_2 _for 12 h (*p < 0.05; **p < 0.01).

A short-term severe hypoxia regime (1% O_2 _for 1 h, 3 h and 4 h) resulted in a significant, gradual decrease of *dmeglob2 *mRNA levels to about 45% of the normoxic control (Figure 5A). Long-term moderate hypoxia (6% O_2 _for 24 h) triggered only a slight down-regulation of *dmeglob2 *mRNA expression (Figure 5B). *Dmeglob3 *mRNA expression was even more reduced (to 23% after 1% O_2 _for 3 h; Figure 5D). After long-term hypoxia (6% O_2 _for 24 h), *dmeglob3 *mRNA level decreased to about 60% compared to the normoxic control (Figure 5E). After hyperoxic treatment, no statistically significant changes of mRNA levels of both, *dmeglob2 *and *3 *were detected (Figure 5C+F). A bioinformatical search for hypoxiaresponsive elements (HRE) [26] in the twelve genomes, conducted by the rVISTA program [27]; http://rvista.dcode.org/), did not yield evidence for interspecifically conserved HRE motifs.

## Discussion

### Globin expression in *Drosophila *testes: a novel or traditional function?

Since the identification of a globin gene (*glob1*) in *D. melanogaster *[13] it has become clear that globins belong to the standard repertoire of most, if not all insects, including those species living in normoxic environments [9]. A conserved common feature of the insect globins studied so far in more detail was their predominant expression in cells of the tracheal system and in the fat body [9,14,18]. Here we convincingly show that the two additional globin genes in the *D. melanogaster *genome, *glob2 *and *glob3 *[18], and also their single counterpart *glob2/3 *in *D. virilis *display an exceptional expression pattern, being almost exclusively transcribed in the adult male reproductive tract during several phases of spermatogenesis. Our qPCR and mRNA *in situ *hybridization results agree with a recent microarray study by Vibranovski et al. [28], which reported a male-specific expression of *dmeglob2 *and *3*. In fact, that study suggested that *dmeglob3 *expression is stronger in post-meiotic compared to meiotic cell populations, whereas *dmeglob2 *exhibits a signal distribution characteristic for genes transcribed during meiosis.

Although globins are traditionally known as O_2 _supply proteins of the respiratory system, the expression of globins in the male reproductive system is not an entirely new fact. The vertebrate neuroglobin, for example, which is well recognized for its cell-protective role in nerve cells, is also expressed for yet unknown reasons in spermatogonia and primary spermatocytes of mouse testes [29]. Very recently, we reported the identification of a highly specialized chimeric protein of yet unknown function, in which a globin domain is fused to a protease-like domain [30]. This chimeric globin was named androglobin due to its predominant expression in testes tissue. While this new member of the globin family is evolutionary ancient and extremely conserved, being present in vertebrates, other chordates, lophotrochozoa, ecdysozoa, more basal animal clades and even choanoflagellates, the available genomes of *Drosophila *do not contain an androglobin ortholog [30]. It is therefore tempting to speculate that flies have evolved the *glob2 *and *glob3 *genes to compensate for a loss of their androglobin lineage. Since glob2 and glob3 are single-domain globins without a fused protease part, they would under this scenario have to interact via their N- and C-terminal extensions [18] with other partner proteins to mimic the role of androglobin.

With the findings for neuroglobin, androglobin and *Drosophila *glob2 and glob3, the expression of globins in the male reproductive system and, more specifically, in the process of spermatogenesis appears to reflect an important aspect of globin function. Over the last decade, the traditional globin functions in O_2 _supply and storage [31,32] have been complemented by other, equally important physiological roles, e.g. in the detoxification of harmful reactive oxygen species [ROS; [33-35]], in the scavenging and/or production of the bio-active gas nitric oxide [NO; [36,37]], in redox-mediated cell signaling and apoptosis regulation [38] and in lipid metabolism [39,40]. Currently, we have only indirect data to distinguish between these possibilities for glob2 and glob3. However, we consider a conventional role of glob2 and glob3 in O_2 _supply in testes rather unlikely in light of the observed transcriptional down-regulation of both genes after experimental hypoxia, while traditional hemoglobins like those in the midge *Chironomus *or the crustacean *Daphnia magna *increase expression under hypoxic conditions [8,41,42]. Alternatively, *Drosophila *glob2 and glob3 may be instrumental in alleviating oxidative stress in the male reproductive tract, which is routinely exposed to ROS formed as by-products during oxidative metabolism. Spermatozoa are particularly sensitive to oxygen-induced damage mediated by lipid peroxidation [43,44]. This sensitivity is enforced by the shortage of antioxidants in the spermatozoa [45,46]. The importance of an effective antioxidants system for correct spermatogenesis in *Drosophila *has been shown e.g. in male flies with a null mutation in the gene for Cu-Zn superoxide dismutase, which leads to a reduced fertility among other phenotypic abnormalities [47,48]. While exposure of flies to oxidative stress was accompanied by an upregulation of antioxidant genes [49], we could not detect such a response for *dmeglob2 *and *3 *after experimental hyperoxia. Thus, currently no correlative evidence for an involvement of glob2 and glob3 in ROS detoxification exists. Modulation of the glob2 and 3 activity by transgenic overexpression or RNAi-mediated knockdown *in vivo *will be instrumental in testing these functional hypotheses. Unfortunately, biophysical data on the ligand binding characteristics of the two proteins are still lacking, because recombinant expression was not successful up to now due to the tendency of both *D. melanogaster *glob2 and glob3 to form stable precipitates. The primary sequences of both globins, however, harbour all key residues required for heme- and gas ligand-binding [18].

### Molecular evolution of the *Drosophila *globin gene family: a complex scenario of neo- and subfunctionalization?

The highly specialized expression pattern of *glob2 *and *glob3 *has to be interpreted in the light of the evolutionary history of the *Drosophila *globin gene family. With the notable exception of the very large globin gene family of chironomids, which provides the midge larvae with huge amounts of extracellular globins for survival in hypoxic aquatic habitats, all other insect taxa studied so far on the genome sequence level have only one (*B. mori*), two (*A. mellifera, A. aegypti, A. gambiae*) or three (*Drosophila *spec.) globin genes [9,12]. Molecular phylogenetic reconstructions based on globin amino acid sequence data have shown that *Drosophila *glob1 forms a clade with the globins of chironomids and the horse botfly *G. intestinalis *[18]. Glob1 is thus representative for a class of presumably O_2_-supplying globins from brachyceran and nematoceran dipterans. In contrast, glob2 and glob3 appeared at a basal position in the globin phylogenetic tree, with no clear affiliations to other insect globins [18]. Evolutionary rate calculations ([18]; this paper) show that glob2 and glob3 have evolved about threefold faster than glob1, so that the ancestral tree position might result from a long-branch attraction artefact. Instead, the absence of *glob2-glob3 *orthologs in other insect genome sequences (our unpublished observations) strongly suggests that this testes-specific globin-type has evolved secondarily in the ancestor of the *Drosophila *genus. This derived evolutionary origin of *glob2-glob3 *would be in agreement with the idea that they compensate for the taxon-specific loss of the testes-expressed androglobin gene lineage.

Gene synteny data enabled us to reconstruct the history of *glob2 *and *3 *within the genus. We identified *glob2 *and *glob3 *orthologs in the nine species, which belong to the *Sophophora *subgenus, whereas the three species of the *Drosophila *subgenus harbour only a single cognate gene (*glob2/3*). Given the widely accepted phylogenetic relations among the twelve species [19], this distribution suggests the duplication of the *glob2/3 *gene in the stem lineage of the *Sophophora *subgenus and thus before the radiation of this clade at least 40 million years ago [20]. This *'glob2/3 *duplication-in-*Sophophora *subgenus' scenario is more parsimonious than the alternative '*glob2 *or *3 *deletion-in-*Drosophila *subgenus' hypothesis, which implies the duplication of a testis-globin ancestor before the radiation of the *Drosophila *genus and the subsequent loss of one of the paralogs in the stem lineage of the *Drosophila *subgenus. Our tree reconstructions and hypothesis testing are fully in line with the more parsimonious interpretation of the *glob2 *and *glob3 *history in the sampled species. The finding of introns in all gene copies suggests that the duplication of the *glob2/3 *gene resulted from unequal crossing over, rather than retrotransposition. Probably, the duplication event in the *Sophophora *ancestor initially resulted in a head-to-tail orientation of *glob2 *and *glob3 *on a chromosome arm, which is equivalent to arm 3R in *D. melanogaster *(event A, Figure 1). This situation is today conserved in *D. ananassae, D. pseudoobscura *and *D. persimilis*. In *D. willistoni *the *glob2 *ortholog has most probably been transposed onto chromosomal arm 2R, equivalent to 2L in *D. melanogaster *(event B, Figure 1). This corresponds to an increased activity of transposable elements in the *D. willistoni *[19]. Likewise, the separation of *glob2 *and *glob3 *by about 800 kb in the representatives of the *D. melanogaster *subgroup probably has been caused by a subsequent transposition of the *glob2 *paralog (event D, Figure 1).

The evolutionary fate of gene duplicates and the consequences of gene duplication for the evolution of novel adaptive traits are matters of intense research and debate [50]. Early studies [e.g. [51]] have contrasted the alternative models of neofunctionalization (i.e. one gene copy stays conserved, the other evolves a novel function) and subfunctionalization (i.e. both gene copies loose part of their functions and have to complement each other). However, ongoing research revealed that evolutionary scenarios of some if not most gene copies might be more complex than anticipated before [52]. Regarding the *glob2 *and *glob3 *gene pair as a possible model case for connecting molecular evolution to the phenotype, we applied codon-based maximum likelihood analyses of sequence evolution, calculating ratios of non-synonymous to synonymous nucleotide substitutions (*d*_N_/*d*_S_), at the codon- and branch-site level to infer selective regimes acting on the globin gene duplicates. The *'glob2/3 *duplication-in-*Sophophora *subgenus' hypothesis, supported by both, parsimony criteria and hypothesis testing, served as the basis for these analyses.

It is well-known that genes encoding proteins involved in sex and reproduction are often subjected to a wide array of selective forces like sexual conflict and male competition [53,54] and actually show signatures of positive selection and adaptive evolution [19,55-59]. Considering an exclusive expression of *D. virilis *and *D. melanogaster glob2 *and *glob3 *in the male genital tract, present evidence from codon-specific analyses for strong negative selection (*d*_N_/*d*_S _= 0.141) of *Drosophila glob2/3, glob2*, and *glob3 *is unexpected and points to high levels of functional constraint. Against this background, it appears remarkable that an estimated proportion of 42% of codon sites experienced strong positive selection (*d*_N_/*d*_S _= 5.642) along the *glob2 *stem lineage (Figure 2). Taking additionally into account the likely absence of a positively selected extra site class along the *glob3 *stem lineage, present observations are not in line with the duplication > degeneration > complementation or subfunctionalization models [51,54-56], nor with the recently introduced model of subneofunctionalization [52]. These models consistently assume similar evolutionary fates of the two gene copies following gene duplication, either as a consequence of relaxed functional constraint [51] or by a succession of relaxed functional constraint and positive selection along both lineages [52]. Present observations rather remind of the classical concept of neofunctionalization *sensu *Ohta [57], assuming the conservation of one gene copy by negative selection, while the paralog evolves a new function under the influence of positive selection. Although the adaptive value of the positively selected amino acid exchanges along the *glob2 *stem lineage has still to be elucidated, results of the present sequence analyses suggest at least some functional differentiation of *Sophophora glob2 *compared to *Sophophora glob3*. On the other hand, the *glob2 *and *glob3 *genes in the *Sophophora *species appear to complement each other in terms of quantitative and regional RNA expression patterns, which is consistent with a subfunctionalization regime. A complex mixture of changing selective constraints over evolutionary time may therefore be a realistic alternative to simple models of gene duplicate evolution.

## Methods

### Sequence retrieval, gene phylogeny, molecular evolution and promoter prediction

Coding sequences of *Drosophila glob2, glob3 *and *glob2/3 *genes were extracted from Flybase http://flybase.org/. Genomic loci were identified by BLASTN search [60], using coding sequences as query. The dataset contained 33 *glob1, glob2, glob3 *and *glob2/3 *sequences of 9 representatives of the *Sophophora *subgenus and 3 representatives of the *Drosophila *subgenus plus *glob1 *of *Gasterophilus intestinalis *and hemoglobin *CTTIII *of *Chironomus thummi thummi *as outgroup representatives. Globin nucleotide sequences were aligned in the amino acid mode using the ClustalX algorithm implemented in BioEdit. For phylogenetic purposes, the highly divergent 5' and 3' ends of the proteins were trimmed, resulting in an alignment of 447 bp, comprising the globin domain.

We tested for potential gene conversion between paralogs at the nucleotide level using Geneconv version 1.81 [61,62], which screens alignments for similar segments in the pairwise comparison suggestive of past gene conversion. To estimate the rates of amino acid sequence evolution of *Drosophila *glob1, glob2 and glob3, pairwise protein distances were calculated using the program MatGat http://bitincka.com/ledion/matgat/ and applying the PAM matrix. We followed the accepted divergence estimates of drosophilid taxa as proposed by Tamura et al. [20].

In order to infer the phylogenetic relations among *glob1, glob2, glob3 *and *glob2/3 *tree reconstructions were carried out at the nucleotide level using a maximum likelihood (ML) approach and Bayesian phylogenetic inference. To minimize the possible influence of saturation on tree reconstruction, only first and second codon positions were used. Applying the Akaike Information Criterion and assuming 4 rate categories, Treefinder [63] identified a special case of the GTR model, called J1[Optimum, Empirical]:G[Optimum]:4 in the Treefinder terminology, as the model of best fit. The J1 model assumes the same rates for i) TA and TG substitutions, and ii) CA and CG substitutions. Except for this constraint, base frequencies, substitution rates and gamma shape parameter were freely estimated from the data. Branch support was estimated by Local Rearrangements of tree support-Expected Likelihood Weights (LR-ELWs) [64] on the basis of 10.000 replicates. The Bayesian analysis was performed using MrBayes v3.1.2 [65,66]. As the J1 model cannot be specified in MrBayes, we assumed the original GTR model instead. We used 2 independent runs, each with four chains and 1.000.000 generations, discarding the first 200.000 generations as 'burnin'. Trees were edited using TreeView [67]. We further elucidated the phylogeny within a clade uniting all *glob2, glob3 *and *glob2/3 *sequences. Therefore, parametric bootstrap testing was performed specifying a topology reflecting the *'glob2/3 *duplication-in-*Sophophora *subgenus' scenario as alternative hypothesis H1 and a topology reflecting the '*glob2 *or *3 *deletion-in-*Drosophila *subgenus' as null hypothesis (H0). At the ortholog level, both trees followed the generally accepted phylogeny within the *Drosophila *genus [19].

We employed the ML framework implemented in CODEML (PAML v4.4 package) [68] to test for signatures of positive selection using the ratio of non-synonymous to synonymous nucleotide substitution rates (ω = *d*_N_/*d*_S_) as a measure. In order to minimize the potential effect of saturation on results, analyses of sequence evolution were carried out based on a restricted dataset of 447 bp comprising *Drosophila glob2/3, Sophophora glob2 *and *Sophophora glob3 *sequences (altogether 21 sequences) and the intree shown in Figure 2. In a first approach, we tested for the presence of candidate codon sites of positive selection across the alignment. In detail, we compared the fit of models M7 (beta) and M8 (beta plus ω) per likelihood ratio test (LRTI). M7 and M8 describe the codon distribution in the ω interval (0, 1) as a beta function. However, while M7 confines ω to (0, 1), M8 allows for a positively selected extra site class. To avoid local optima, we ran M8 twice with different initial ω values (0.6 and 1.6). For LRTI, twice the log likelihood difference (2*Δl*) between the nested models was compared to critical values (cv) from a chi-square distribution with degrees of freedom (df) equal to the difference in the number of free parameters between the models, which is 4-2 = 2.

We moreover tested for the presence of positively selected codon sites across user-defined "foreground" branches (branch-site LRTII). In detail, we compared the fit of two versions of modified branch-site model A [69]. Both model versions assume a negatively selected site class and a neutrally evolving site class across the entire phylogeny. Both versions further distinguish a third site class with *d*_N_/*d*_S _< 1 or *d*_N_/*d*_S _= 1 across the background. However, while *d*_N_/*d*_S _of the third site class is fixed at 1 across the foreground in the null version of model A, the alternative version allows for a foreground *d*_N_/*d*_S _> 1. We alternatively defined the *Sophophora glob2 *and the *Sophophora glob3 *stem lineages as foreground branches. For LRTII, 2*Δl *between the two model versions was compared to cv from a 50:50 mixture of a point mass at zero and a chi-square distribution with *df *= 1, which are 2.71 and 3.84 at the 5% and 2.5% levels of significance. Strict Bonferroni adjustment was performed to adjust for twofold testing, thus lowering the 5% level of significance to 2.5%.

The identification of possible binding-sites of RNA polymerase II was performed with the program Neural Network Promoter Prediction (NNPP) on the Berkeley *Drosophila *Genome Project homepage http://www.fruitfly.org/seq_tools/promoter.html. The NNPP program recognizes RNA polymerase II promoter via a TATA box and the Initiator transcription start site [23,24]. Additionally, we used the hidden Markov model-based program McPromoter http://tools.igsp.duke.edu/generegulation/McPromoter/[21,22] with the genomic sequences of *D. melanogaster glob2, glob3*, and *D. simulans *and *D. sechellia glob3 *as input.

### Fly stocks

Hypoxia experiments were carried out using *D. melanogaster *wild type strain Oregon R. *In situ *hybridization and developmental stage-specific *glob2, glob3 *and *glob2/3 *mRNA expression analyses were carried out using *D. melanogaster *wild type strain Oregon R and *D. virilis*. Flies were kept on standard cornmeal agar at 25°C.

### Hypoxia and hyperoxia experiments

25 adult *D. melanogaster *were experimentally exposed to moderate hypoxia (5% O_2 _for 24 h), severe hypoxia (1% O_2 _for 1 h, 3 h and 4 h) and hyperoxia (95% O_2 _for 12 h), using a translucent PRO-OX chamber (BioSpherix Ltd., New York, USA). During hypoxia treatments, animals were monitored for vitality and behavioural reactions that are known being caused by the applied O_2 _concentrations (see e.g. [70]. Flies were kept at 25°C at the pre-adjusted O_2 _concentration that were obtained by mixing nitrogen or oxygen with ambient air. Technical nitrogen and oxygen were obtained from Westfalen AG (Münster, Germany). Gas concentrations were measured and kept constant by an oxygen sensor (E-702, BioSpherix, Ltd., New York, USA). After the desired time, animals were collected and immediately shock-frozen in liquid nitrogen. Samples were stored at -80°C until use.

### RNA preparation and quantitative real-time reverse transcription-PCR (qPCR)

Total RNA was isolated using the RNeasy Mini Kit (Qiagen, Hilden, Germany), quantified and checked on integrity. Reverse transcription was carried out with 1 μg total RNA employing the Superscript II RNase H^- ^reverse transcriptase (Invitrogen, Karlsruhe, Germany). qPCR experiments were carried out on the ABI Prism 7500 Sequence Detection System (Applied Biosystems, Darmstadt, Germany). For primer sequences, see Additional File 5. mRNA expression levels were calculated by the standard-curve approach, measuring the Ct-values. Factors of differential gene expression in developmental stages were calculated relative to the gene expression in adult males. *D. melanogaster glob2, glob3 *and *lactate dehydrogenase (LDH) *expression data under different oxygen conditions were normalized relative to expression of the ribosomal protein gene *L17A*, which is not regulated by hypoxia according to microarray experiments (B. Adryan and R. Schuh, Göttingen, personal communication). Factors of differential gene regulation were calculated relative to the normoxic condition (21% O_2_). Statistical evaluation was performed by calculating the mean values of the factors of regulation and their standard deviation. Two independent experiments (biological replicates) were performed for each condition, and each qPCR assay was run in duplicate. The significance of the data was assessed by a two-tailed Student's *t*-test employing the Microsoft Excel spreadsheet program.

### Preparation of genomic DNA and genomic PCR

Genomic DNA was isolated from 20 L3 larvae according to the protocol by Huang et al. [71]. Genomic PCR was performed using Taq polymerase (Sigma-Aldrich Chemie GmbH, Munich, Germany) according to the manufacturer's recommendations (for primer sequences, see Additional File 7).

### mRNA *in situ *hybridization

mRNA probes for *in situ *hybridization experiments were prepared by *in vitro *transcription from the coding sequence of *D. melanogaster glob2, glob3 *and *D. virilis glob2/3 *using the digoxigenin RNA Labelling Kit (Roche Applied Science, Mannheim, Germany). The *D. melanogaster *β-tubulin 85D was used as a positive control for testes-specific mRNA expression [72]. It revealed the expected staining of cells in meiosis II (not shown). *In situ *hybridization to whole testes was carried out according to the protocol from Tautz and Pfeifle [73] with the following changes: testes were dissected in testes buffer [74-76], fixed with 4% formaldehyde/PBS for 30 min and washed briefly in PBS + 0.1% (v/v) Tween-20. Tissues were pre-incubated with hybridization solution (50% [v/v] 10x Diethylpyrocarbonate-SSC, 50% [v/v] formamide, 0.1% [v/v] Tween-20) containing 0.1 mg/ml salmon sperm carrier DNA at 55°C. Hybridization was carried out at 55°C overnight in hybridization solution containing 0.1 mg/ml salmon sperm DNA and 0.5 μg/mL antisense probe. Washing was performed in PBS + 0.1% (v/v) Tween-20 at 65°C. Detection and staining were carried out using an anti-DIG alkaline phosphatase-conjugated antibody and NBT/BCIP solution (Roche Applied Science, Mannheim, Germany) according to the manufacturer's recommendations.

## Authors' contributions

EG participated in the design of the study, carried out qPCR and mRNA *in situ *hybridization experiments, extracted the *glob2, glob3 *and *glob2/3 *genes in the available sequenced *Drosophila *genomes and analyzed their genomic organization, calculated the evolutionary rates, performed the promoter search, participated in the sequence alignments, phylogenetic reconstructions and in the analyses regarding molecular evolution and drafted the manuscript. HH participated in the sequence alignments, phylogenetic reconstructions, in the analyses regarding molecular evolution and helped to draft the manuscript. SZ participated in qPCR and mRNA *in situ *hybridization experiments and helped to draft the manuscript. TB helped to draft the manuscript. TH conceived the study, participated in its design and coordination, and helped to draft the manuscript. All authors read and approved the final manuscript.

## Supplementary Material

Additional file 1 **Designations of *Drosophila *globin genes**. Click here for file

Additional file 2 **Phylogenetic relationship of *Drosophila *globins**. Phylogenetic reconstruction of *Drosophila glob1, glob2, glob3 *and *glob2/3 *including *G. intestinalis glob1 *(ginglob1) and *C. thummi thummi HbIII *(cttHbIII) at the nucleotide level (using only first and second codon positions). (A) by applying a Maximum likelihood approach implemented in Treefinder and (B) a Bayesian analysis using MrBayes. Click here for file

Additional file 3 **Absolute mRNA quantitation of *dmeglob2, dmeglob3 *and *dviglob2/3***. *Glob2, glob3 *and *glob2/3 *absolute mRNA copy number in male adult flies of *D. melanogaster *and *D. virilis*, measured with qPCR. *Dmeglob2 *(144784 copies) and *dmeglob3 *(150026 copies) copy number summed up is equal to *dviglob2/3 *copy number (311673 copies). Click here for file

Additional file 4 ***Glob3 *gene region with Jockey transposon**. Schematic diagram of the genomic region of *D. melanogaster glob3 *including the transposable element inserted downstream of the *glob3 *gene in comparison to the corresponding genomic region of *D. sechellia glob3*. In *D. melanogaster*, the insertion and the putative duplicated sequences are indicated. In *D. sechellia*, the predicted promoter sequence spanning the transposon insertion sequence in *D. melanogaster *are highlighted. Exons and 5'UTR and distances between 3'end of transposable element and Exon1 in both *D. melanogaster *and *D. sechellia *are plotted. Click here for file

Additional file 5 **Negative control for mRNA *in situ *hybridization**. As negative control for *in situ *hybridization, a sense mRNA probe of dmeglob2 was applied at otherwise identical hybridization conditions. Click here for file

Additional file 6 **Regulation of *LDH *mRNA in *D. melanogaster *male adults after hypoxic stress**. mRNA levels (bars) are shown relative to the gene expression at normoxia (21%). (A) *LDH *expression after 1% O_2 _for 1 h, 3 h and 4 h. After 1 h of hypoxia, no alteration in *LDH *expression could be detected. 3 h of hypoxia caused the *LDH *mRNA levels to increase about 2.2 fold and 4 h of hypoxia to about 3.4 fold compared to the normoxic control (B) *LDH *expression after 6% O_2_for 24 h. After applying long-term moderate hypoxia with 6% O_2 _for 24 h, an increase in *LDH *mRNA expression to about 1.5 fold could be detected (*p < 0.05; **p < 0.01). Click here for file

Additional file 7 Oligonucleotides and PCR conditions Click here for file
