# Supplementary material for: Testes-specific hemoglobins in Drosophila evolved by a combination of sub- and neofunctionalization after gene duplication
Source: BMC Evol Biol. 2012 Mar 19;12:34. doi: 10.1186/1471-2148-12-34 (PMC3361466; doi:10.1186/1471-2148-12-34)
Supplement: Additional file 1 — Designations of Drosophila globin genes. [file 1471-2148-12-34-S1.PDF]

Additional File 1: Sequences used in this study

Species		Annotation ID
D. melanogaster glob1	(dmeglob1)	CG9734
D. melanogaster glob2	(dmeglob2)	CG15180
D. melanogaster glob3	(dmeglob3)	CG14675
D. simulans glob1	(dsiglob1)	GD20318
D. simulans glob2	(dsiglob2)	GD19540
D. simulans glob3	(dsiglob3)	GD19828
D. sechellia glob1	(dseglob1)	GM25744
D. sechellia glob2	(dseglob2)	GM10544
D. sechellia glob3	(dseglob3)	GM10846
D. erecta glob1	(derglob1)	GG20278
D. erecta glob2	(derglob2)	GG10391
D. erecta glob3	(derglob3)	GG13071
D. yakuba glob1	(dyaglob1)	GE26348
D. yakuba glob2	(dyaglob2)	GE24095
D. yakuba glob3	(dyaglob3)	GE10172
D. ananassae glob1	(danglob1)	GF16470
D. ananassae glob2	(danglob2)	GF16358
D. ananassae glob3	(danglob3)	GF16359
D. pseudoobscura glob1	(dpsglob1)	GA21995
D. pseudoobscura glob2	(dpsglob2)	GA26482
D. pseudoobscura glob3	(dpsglob3)	GA26483
D. persimilis glob1	(dpeglob1)	GL21726
D. persimilis glob2	(dpeglob2)	GL24031
D. persimilis glob3	(dpeglob3)	GL24032
D. willistoni glob1	(dwiglob1)	GK11857

D. willistoni glob2	(dwiglob2)	GK24567
D. willilstoni glob3	(dwiglob3)	GK14442
D. mojavensis glob1	(dmoglob1)	GI24641
D. mojavensis glob3	(dmoglob3)	GI23233
D. virilis glob1	(dviglob1)	GJ23463
D. virilis glob3	(dviglob3)	GJ22893
D. grimshawi glob1	(dgrglob1)	GH13816
D. grimshawi glob3	(dgrglob3)	GH18925
G. intestinalis glob1	(ginglob1)	AF063938
C. thummi thummi hbIII	(ctthbIII)	P02229
