# Supplementary material for: Testes-specific hemoglobins in Drosophila evolved by a combination of sub- and neofunctionalization after gene duplication
Source: BMC Evol Biol. 2012 Mar 19;12:34. doi: 10.1186/1471-2148-12-34 (PMC3361466; doi:10.1186/1471-2148-12-34)
Supplement: Additional file 2 — Phylogenetic relationship of Drosophila globins. Phylogenetic reconstruction of Drosophila glob1, glob2, glob3 and glob2/3 including G. intestinalis glob1 (ginglob1) and C. thummi thummi HbIII (cttHbIII) at the nucleotide level (using only first and second codon positions). (A) by applying a Maximum likelihood approach implemented in Treefinder and (B) a Bayesian analysis using MrBayes. [file 1471-2148-12-34-S2.PDF]

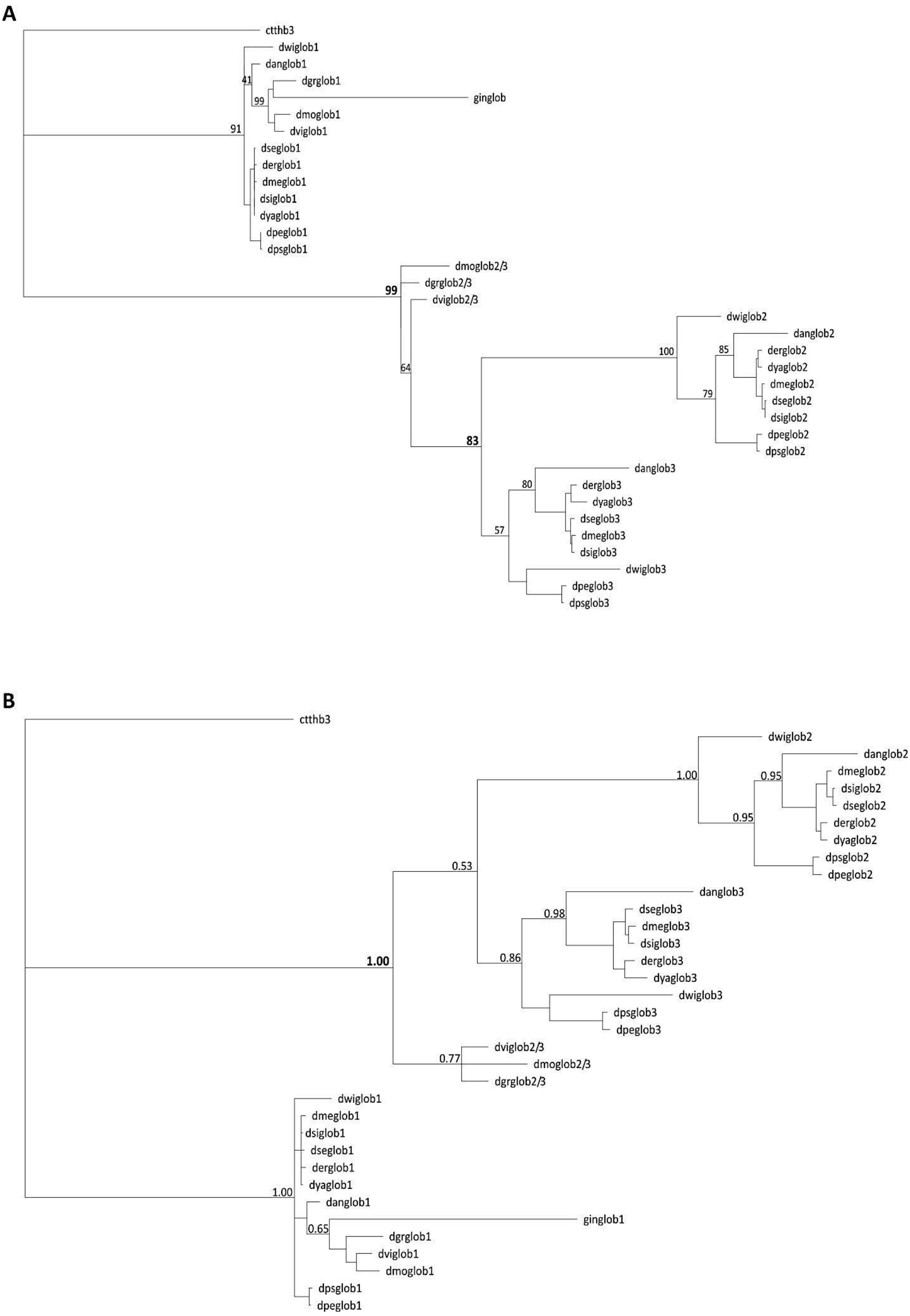
A
ctthb3
dwiglob1
danglob1
41
dgrglob1
ginglob
99
dmoglob1
91
dviglob1
dseglob1
derglob1
dmeglob1
dsiglob1
dyaglob1
dpeglob1
dpsglob1
dmoglob2/3
99
dgrglob2/3
dviglob2/3
dwiglob2
danglob2
100
85
derglob2
dyaglob2
64
dmeglob2
79
dseglob2
dsiglob2
dpeglob2
83
dpsglob2
danglob3
80
derglob3
dyaglob3
dseglob3
57
dmeglob3
dsiglob3
dwiglob3
dpeglob3
dpsglob3
B
ctthb3
dwiglob2
danglob2
1.00
0.95
dmeglob2
dsiglob2
dseglob2
0.95
derglob2
dyaglob2
dpsglob2
0.53
dpeglob2
danglob3
0.98
dseglob3
dmeglob3
dsiglob3
1.00
derglob3
0.86
dyaglob3
dwiglob3
dpsglob3
dpeglob3
dviglob2/3
0.77
dmoglob2/3
dgrglob2/3
dwiglob1
dmeglob1
dsiglob1
dseglob1
derglob1
dyaglob1
1.00
danglob1
ginglob1
0.65
dgrglob1
dviglob1
dmoglob1
dpsglob1
dpeglob1

Additional File 2: Phylogenetic relationship of *Drosophila* globins

Phylogenetic reconstruction of *Drosophila glob1*, *glob2*, *glob3* and *glob2/3* including *G. intestinalis glob1* (*ginglob1*) and *C. thummi thummi hbIII* (*ctthbIII*) at the nucleotide level (using only first and second codon positions). (A) by applying a Maximum likelihood approach implemented in Treefinder and (B) a Bayesian analysis using MrBayes.
