# Supplementary material for: Testes-specific hemoglobins in Drosophila evolved by a combination of sub- and neofunctionalization after gene duplication
Source: BMC Evol Biol. 2012 Mar 19;12:34. doi: 10.1186/1471-2148-12-34 (PMC3361466; doi:10.1186/1471-2148-12-34)
Supplement: Additional file 3 — Absolute mRNA quantitation of dmeglob2, dmeglob3 and dviglob2/3. Glob2, glob3 and glob2/3 absolute mRNA copy number in male adult flies of D. melanogaster and D. virilis, measured with qPCR. Dmeglob2 (144784 copies) and dmeglob3 (150026 copies) copy number summed up is equal to dviglob2/3 copy number (311673 copies). [file 1471-2148-12-34-S3.PDF]

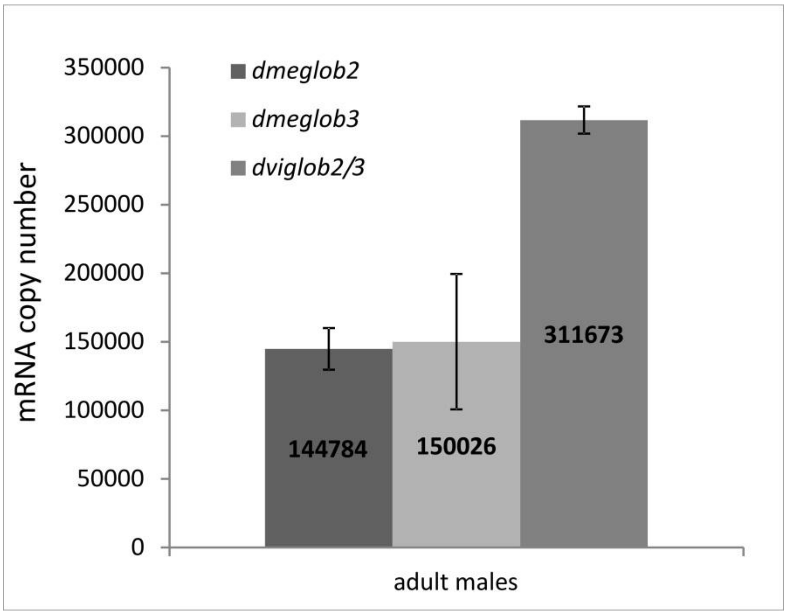

Additional File 3: Absolute mRNA quantitation of *dmeglob2*, *dmeglob3* and *dviglob2/3*

Glob2, glob3 and *glob2/3* absolute mRNA copy number in male adult flies of *D. melanogaster* and *D. virilis*, measured with qPCR. *Dmeglob2* (144784 copies) and *dmeglob3* (150026 copies) copy number summed up is equal to *dviglob2/3* copy number (311673 copies).
