# Supplementary material for: Testes-specific hemoglobins in Drosophila evolved by a combination of sub- and neofunctionalization after gene duplication
Source: BMC Evol Biol. 2012 Mar 19;12:34. doi: 10.1186/1471-2148-12-34 (PMC3361466; doi:10.1186/1471-2148-12-34)
Supplement: Additional file 4 — Glob3 gene region with Jockey transposon. Schematic diagram of the genomic region of D. melanogaster glob3 including the transposable element inserted downstream of the glob3 gene in comparison to the corresponding genomic region of D. sechellia glob3. In D. melanogaster, the insertion and the putative duplicated sequences are indicated. In D. sechellia, the predicted promoter sequence spanning the transposon insertion sequence in D. melanogaster are highlighted. Exons and 5'UTR and distances between 3'end of transposable element and Exon1 in both D. melanogaster and D. sechellia are plotted. [file 1471-2148-12-34-S4.PDF]

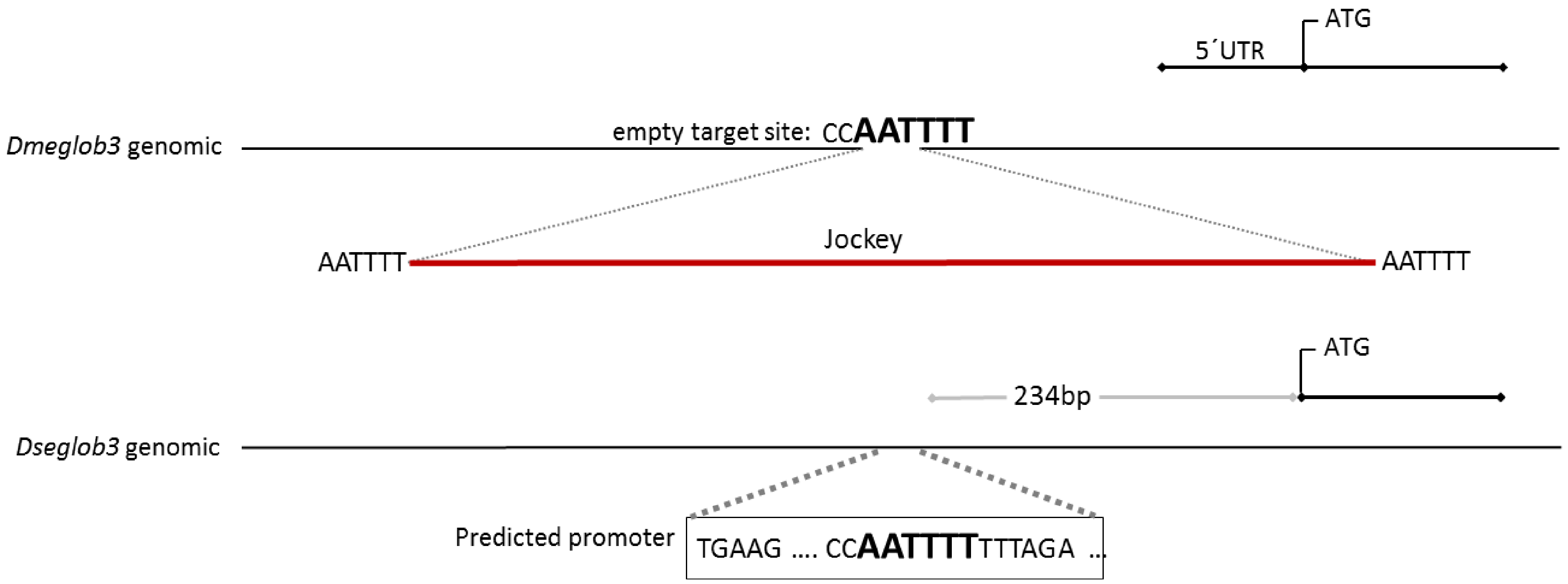

Additional File 4: *Glob3* gene region with Jockey transposon

Schematic diagram of the genomic region of *D. melanogaster glob3* including the transposable element inserted downstream of the *glob3* gene in comparison to the corresponding genomic region of *D. sechellia glob3*. In *D. melanogaster*, the insertion and the putative duplicated sequences are indicated. In *D. sechellia*, the predicted promoter sequence spanning the transposon insertion sequence in *D. melanogaster* are highlighted. Exons and 5´UTR and distances between 3´end of transposable element and Exon1 in both *D. melanogaster* and *D. sechellia* are plotted.
