# Supplementary material for: Testes-specific hemoglobins in Drosophila evolved by a combination of sub- and neofunctionalization after gene duplication
Source: BMC Evol Biol. 2012 Mar 19;12:34. doi: 10.1186/1471-2148-12-34 (PMC3361466; doi:10.1186/1471-2148-12-34)
Supplement: Additional file 5 — Negative control for mRNA in situ hybridization. As negative control for in situ hybridization, a sense mRNA probe of dmeglob2 was applied at otherwise identical hybridization conditions. [file 1471-2148-12-34-S5.PDF]

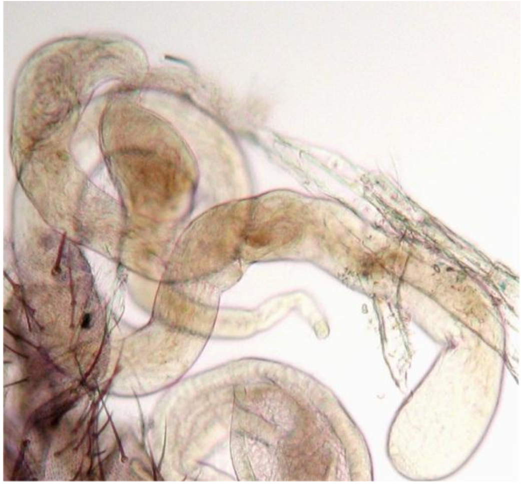

Additional File 5: Negative control for mRNA *in situ* hybridization

As negative control for the *in situ* hybridization, a sense mRNA probe of *dmeglob2* was applied at otherwise identical hybridization conditions.
