# Supplementary material for: Testes-specific hemoglobins in Drosophila evolved by a combination of sub- and neofunctionalization after gene duplication
Source: BMC Evol Biol. 2012 Mar 19;12:34. doi: 10.1186/1471-2148-12-34 (PMC3361466; doi:10.1186/1471-2148-12-34)
Supplement: Additional file 6 — Regulation of LDH mRNA in D. melanogaster male adults after hypoxic stress. mRNA levels (bars) are shown relative to the gene expression at normoxia (21%). (A) LDH expression after 1% O2 for 1 h, 3 h and 4 h. After 1 h of hypoxia, no alteration in LDH expression could be detected. 3 h of hypoxia caused the LDH mRNA levels to increase about 2.2 fold and 4 h of hypoxia to about 3.4 fold compared to the normoxic control (B) LDH expression after 6% O2for 24 h. After applying long-term moderate hypoxia with 6% O2 for 24 h, an increase in LDH mRNA expression to about 1.5 fold could be detected (*p < 0.05; **p < 0.01). [file 1471-2148-12-34-S6.PDF]

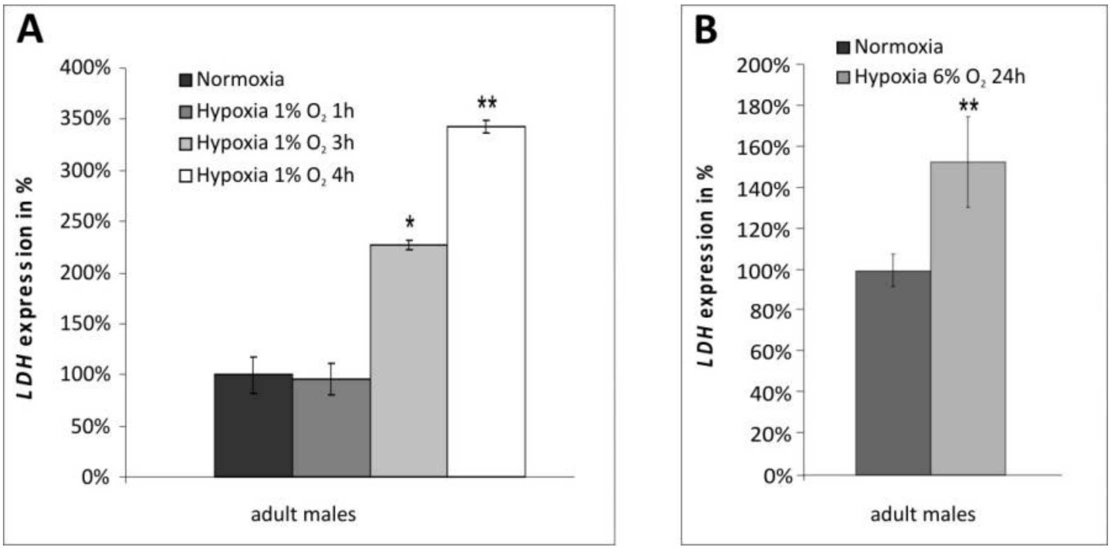

Additional File 6: Regulation of *LDH* mRNA in *D. melanogaster* male adults after hypoxic stress

mRNA levels (bars) are shown relative to the gene expression at normoxia (21%). (A) *LDH* expression after 1% O_2 for 1 h, 3 h and 4 h. After 1 h of hypoxia, no alteration in *LDH* expression could be detected. 3 h of hypoxia caused the *LDH* mRNA levels to increase about 2,2 fold and 4 h of hypoxia to about 3,4 fold compared to the normoxic control (B) *LDH* expression after 6 % O_2 for 24 h. After applying long-term moderate hypoxia with 6 % O_2 for 24 h, an increase in *LDH* mRNA expression to about 1,5 fold could be detected (*$p < 0.05$; **$p < 0.01$).
