# Supplementary material for: Testes-specific hemoglobins in Drosophila evolved by a combination of sub- and neofunctionalization after gene duplication
Source: BMC Evol Biol. 2012 Mar 19;12:34. doi: 10.1186/1471-2148-12-34 (PMC3361466; doi:10.1186/1471-2148-12-34)
Supplement: Additional file 7 — Oligonucleotides and PCR conditions [file 1471-2148-12-34-S7.PDF]

Additional File 7: Oligonucleotides and PCR conditions

For QPCR experiments we used the following oligonucleotide primer combinations: *dmeglob2* 5´- ata tga gga tgc tgg cc acc -3´ and 5´- cgt caa cag tcg tcc taa gg -3´; *dmeglob3* 5´- gtt aat att ggg cat ctg gcc -3´ and 5´- gta ttc gac tgc tgg tcc tg -3´; *dviglob2/3* 5´- gag cat atg agg atg ttg atg aac -3´ and 5´- gcg aag tct gag caa aga tag c -3´; *L17A* 5´- taa cca gtc cgc gag cag c -3´ and 5´- gtc atg cct gcc gtg gtt att -3´; *LDH*, 5'- cta aca gat cca ttc gca aca cc -3' and 5'- act tga tgc tac gat tcg tgg -3'. The gene for ribosomal protein *L17A* was used as a reference for normalization, the gene for lactate dehydrogenase *LDH* served as a positive control for hypoxia-induced changes in gene expression. After activation of the polymerase at 95°C for 15 min, amplification was performed in a three-step protocol: 94°C for 15 sec, 60°C for 30 sec, 72°C for 30 sec, measuring the fluorescence during the last step of each cycle. All PCR experiments were followed by dissociation curves at a temperature range from 60°C to 95°C to analyse the specificity of the amplification reactions. No unspecific products or primer dimers were detected by melting curve analysis and gel electrophoresis of PCR amplificates.

For detection of the Jockey transposable element the following oligonucleotide primer were used: *dmeglob3* Jockey 5´-ttg acc ttc cgt tgg tgt ttg-3´ and 5´-ttg ggt agg taa gac ttg aca ag-3´.
